# Translating Research into Practice in Low-Resource Countries: Progress in Prevention of Maternal to Child Transmission of HIV in Nigeria

**DOI:** 10.1155/2013/848567

**Published:** 2013-04-29

**Authors:** Y. Ogbolu, E. N. Iwu, S. Zhu, J. V. Johnson

**Affiliations:** ^1^Office of Global Health, University of Maryland School of Nursing, 655 W. Lombard Street, Baltimore, MD 21201, USA; ^2^University of Maryland School of Nursing, Baltimore, MD 21201, USA; ^3^Institute of Human Virology, Pent House, Maina Court, Plot 252, Herbert Macaulay Way, Central Business District, P.O. Box 9396, Garki, Abuja, Nigeria

## Abstract

*Background.* Research related to prevention of maternal to child transmission (PMTCT) of HIV is dynamic and rapidly changing and has provided evidence-based interventions and policies for practitioners. However, it is uncertain that research and policy guidelines are adequately being disseminated and implemented in resource-constrained countries with the largest burden PMTCT. This study examined current PMTCT practices in 27 public health facilities in Nigeria. *Methods.* A cross-sectional survey of 231 practicing nurses was conducted. Current PMTCT care practices were evaluated and compared to WHO and national PMTCT policy guidelines. Linear mixed models evaluated the association between PMTCT care practices and training in PMTCT. *Results.* Most nurses (80%) applied practices involving newborn prophylaxis; yet significant gaps in maternal intrapartum treatment and infant feeding practices were identified. PMTCT training explained 25% of the variance in the application of PMTCT care practices. *Conclusion.* Key PMTCT practices are not being adequately translated from research into practice. Researchers, policymakers, and clinicians could apply the study findings to address significant knowledge translation gaps in PMTCT. Strategies derived from an implementation science perspective are suggested as a means to improve the translation of PMTCT research into practice in Sub-Saharan African medical facilities.

## 1. Background

Each year, an estimated 350,000 infants, mostly in low-resource countries, acquire human immunodeficiency virus infection from their mothers [1, 2]. Resource-limited countries in Sub-Saharan Africa continue to bear the greatest burden of maternal to child transmission and account for the highest number of new pediatric human immunodeficiency virus (HIV) infections globally. Nigeria alone is responsible for 30% of the global burden of maternal to child transmission of HIV and has joined a group of 22 countries as part of a global initiative to reduce the number of new pediatric HIV infections [3]. Prevention of maternal to child transmission (PMTCT) has become a key public health priority in Nigeria, a country faced with 56,681 annual HIV-positive births and more than 210,000 women living with HIV [2, 4]. In high-resource countries, successful implementation of evidence-based interventions from research has resulted in a reduction of perinatal HIV infections to 2% or less [5, 6]. If evidence from current international studies is translated successfully from research into practice in resource-limited countries, the burden of maternal to child transmission of HIV may be reduced. Global and national guidelines have been developed using this international evidence; yet there has been limited research examining whether or not this evidence has been applied within clinical practice settings in resource-limited countries. Enabling the translation of evidence-based practice from research to frontline nursing is a critical element of a systems approach to reducing maternal to child transmission of HIV.

Registered nurses make up more than 60% of the healthcare workforce in Sub-Saharan Africa [7, 8] and serve as the backbone of the healthcare system, according to the World Health Organization (WHO) [9]. In many African countries, including Nigeria, nurses are dually certified as midwives, which places them in a position to prevent maternal to child transmission of HIV. Given this dual role, nurse/midwife and nurse are used interchangeably throughout the paper. This critically important role extends from preconception to prenatal, perinatal, postnatal, gynecologic, and family planning. The specific nursing practices aimed at eliminating maternal to child transmission of HIV across phases of the childbearing period are outlined in Table 1.

Task shifting of HIV care and treatment responsibilities from physicians to nurses has resulted in an expansion of the nurse's role as the primary medical care provider for mothers with HIV and AIDS in many resource-limited regions of the world [10–12]. When task-shifting practices are well implemented, nurses and midwives in rural, hard-to-reach communities are empowered to provide appropriate, integrated, patient-centered PMTCT services. Research has demonstrated that lay health workers, including traditional birth attendants (TBAs), directly providing health services to mothers and children depend on nurse/midwives as their most common source of information related to PMTCT of HIV [13]. Given that 60% of women in Nigeria continue to be delivered by lay workers who rely on governmental nurses/midwives for their knowledge related to PMTCT, it is important that the nurses/midwives offer the best advice. In view of the current global agenda for an AIDS-free generation, it is essential that frontline maternal, newborn, child health (MNCH) nurses in resource-limited settings have the knowledge and ability to utilize the best evidence-based practices in PMTCT.

Despite nursing's important role, research has not adequately examined whether evidence-based knowledge is being translated into the practice settings of nurses in resource-limited settings, like Nigeria. The purpose of the present study is to examine the translation of evidence-based practices related to PMTCT in maternal, newborn, child health nurse/midwives (*n* = 231) in 27 public health facilities in Nigeria. The paper begins with a review of studies that examine HIV knowledge and practice among nurses in developing countries. Next, the methods and results of the study are presented, followed by a discussion of study implications related to the challenges nurses face in the translation of evidence-based practices in perinatal HIV prevention in resource-limited settings. The conclusion applies an implementation science perspective to formulate recommendations that could enhance the translation of PMTCT research to nursing practice.

### 1.1. Review of the Literature

An extensive literature review resulted in a limited number of articles directly related to the translation of PMTCT evidence-based practices among nurses. The literature review was subsequently expanded to include studies examining the HIV/AIDS knowledge and practice of other types of MNCH providers in developing countries. Multiple studies in Sub-Saharan Africa have indicated gaps in nursing knowledge associated with minimal access to training in HIV/AIDS care [14, 15]. Research on nurses has identified specific gaps in knowledge, including the inability to identify high-risk groups, describe symptoms, interpret diagnostic tests, and utilize universal precautions. Studies on nursing populations have also reported decreased confidence in their knowledge of HIV, inadequate understanding of appropriate infant feeding practices, and limited skills in HIV counseling and assessment of medication adherence [14]. Clinical experience, frequency of care, and greater levels of training have been shown to be associated with significant improvements in knowledge of HIV/AIDS care among nurses [15]. Research studies specifically examining PMTCT knowledge in nurses are quite limited. A recent Nigerian study examined PMTCT practice in traditional birth attendants (TBAs) and found that the overwhelming majority (91%) lacked basic knowledge related to HIV/AIDS [13]. TBAs reported performing unsafe PMTCT care practices, which limited their ability to prevent HIV in mothers, reduce transmission to newborns, and safely protect themselves from HIV. Nurses may assist in the reduction of unsafe practices related to PMTCT by providing TBAs with reliable information. The marked scarcity of studies on translation of PMTCT knowledge into the practice setting indicates the need for further research in this area.

## 2. Methods

### 2.1. Design

The study employed a cross-sectional survey design of maternal, newborn, child health nurses across 27 public healthcare institutions in Nigeria.

### 2.2. Theoretical Framework

The diffusions of innovation theory [16, 17] was used as the conceptual framework for this paper. This model has been used extensively to examine the transfer of evidence-based knowledge from research to policy and practice. The model identifies five core components that determine whether a new evidence-based practice is adopted and subsequently diffused into practice. First, *relative advantage* must be present; that is, key practitioners must perceive the new evidence-based practice as an improvement to the current practice. The second component is *compatibility*, which considers whether the new practice works well with existing practices, past experiences, and the needs of the potential end users. *Complexity* is the third component, which considers whether the practice is relatively simple and well defined. The newly developed practice must also demonstrate the fourth component, *trialability*, meaning opportunities must be offered for the practice to be tested or trialed in the clinical setting. Finally, *observability* allows nurses to see the evidence-based practice demonstrated by key clinical leaders within their particular clinical setting.

### 2.3. Sample

The sample was drawn from a sample frame of 140 public health facilities supported by the Institute of Human Virology, Nigeria (IHV-N), a major Presidential Emergency Program for AIDS Relief (PEPFAR) implementing partner in Nigeria. Through grants administered by the Center for Disease Control and Prevention in Nigeria, IHV-N provides technical support to the government of Nigeria for HIV/AIDS prevention, diagnosis, treatment, and care and support services at approximately 140 health facilities. These facilities include teaching and general hospitals as well as community-based primary health centers within the six geopolitical zones of Nigeria. A convenience sample of public health facilities across all three tiers of the healthcare system (primary, secondary, and tertiary) was selected and twenty-seven facilities agreed to participate. Facilities were chosen based on government ownership and their geographic location in the northern, southeastern, and south-southern regions of Nigeria. All MNCH nurses at each of the participating facilities were invited to complete the anonymous, self-administered survey, and 231 nurses completed it. Nurses were clustered within facilities, and the response rate was greater than 95% at each facility. Nurses not actively employed in the care of pregnant women or newborns were excluded.

### 2.4. Data Collection

Nigerian nurses from University of Maryland and our collaborating nurses at the Institute of Human Virology in Nigeria both provided face validity and expert validity by reviewing and piloting the nurse survey prior to implementation. Healthcare providers in Nigeria speak English as their official language; thus, language translation of the self-administered survey documents was not necessary. Data collection occurred over 6 weeks from March to May 2010. The survey consisted of the following sections: demographics; knowledge questions related to PMTCT; and a nursing care practices scale related to direct PMTCT nursing care. The surveys were implemented in 27 healthcare facilities across 10 of the 36 states in Nigeria. All maternal, newborn, child health nurses on duty on the day of the in-person administrative interview were invited to participate, and the response rate was 95 percent or greater for each site.

### 2.5. Measures

#### 2.5.1. PMTCT Practice Scale

The PMTCT practice scale consists of 12 items related to nursing practices associated with the prevention of maternal to child transmission. Translation was explored with a series of survey questions, which are aligned with the WHO and Nigerian Federal Ministry of Health (FMOH) guidelines on PMTCT. The items were dichotomous (yes/no), and the scale included evidence-based practices related to postexposure prophylaxis for newborns, newborn feeding practices, maternal prenatal laboratory testing and screening, maternal treatment during delivery, and availability of protective equipment for universal precautions. Cronbach's alpha for the PMTCT practice scale was 0.621, consistent with other HIV knowledge scales [17].

### 2.6. Ethical Considerations

The appropriate ethics committees and health authorities approved the study. The Institutional Review Board of the University of Maryland Baltimore and the Institute of Human Virology Nigeria (IHV-N) Institutional Review Board both approved the study.

### 2.7. Data Analysis

Data were analyzed using SPSS Version 19. Exploratory analysis was used to detect potential outliers and data collection errors. Standard descriptive statistics (mean, standard deviation, and frequency) were used to describe all key variables. During the data collection phase, each nurse survey was coded to indicate the facility in which the nurse respondent practiced. During data cleaning, eight surveys were noted to have >80% missing data and were deleted from the analysis. The remaining surveys (*n* = 223) had less than 10% missing data for all variables and served as the final sample for analyses. Practice-related knowledge in prevention of maternal to child transmission of HIV and the proportion of nurses participating in training were analyzed. Differences in characteristics in groups of nurses receiving PMTCT training were examined using *t*-test or chi-square test. Student's *t*-test was used to examine mean differences in age, nursing experience, and PMTCT knowledge scores. Chi-square or Fischer's exact test (for those with fewer than five in a group) analysis was applied for bivariate analysis of categorical variables, nursing education, professional rank, and unit. Finally, multilevel modeling approaches, that is, bivariate and multivariable linear mixed models, were used to account for clustering of nurses within facilities.

## 3. Results

### 3.1. Sample Characteristics

As depicted in Table 3, nearly half (43%) of the 223 participating nurses had received training in PMTCT. Nurses who received training were significantly older, mean age 43 years (SD 8.1) compared to nontrained, mean  age = 39 (SD 8.8); *t* = −3.3, *P* = 0.001. While intercultural differences between and within the major tribes (Hausa, Ibo, and Yoruba) and geographic regions in Nigeria do exist, respect and privilege for older adults (including nurses) is a culturally bounded practice, which may result in older nurses having more opportunities for training [18, 19]. The nurses were highly experienced, with an average of 16.5 years in nursing and within maternal, newborn, child health nursing, specifically, a mean of 8.7 years. Most nurse participants were diploma-prepared nurses (>90%), and although the group size was small, nurses with a BS or MS degree reported not receiving PMTCT training. Currently 75% of practicing RNs in Nigeria are certificate and diploma holders [20]. All respondents were MNCH nurses and were employed in various specialty units, including 34% in labor and delivery, followed by 17% in the special baby care units, 14.3% in antenatal care/prenatal care clinics, and 14.2% in pediatric units. The largest number of nurses in the study who received PMTCT training were maternity (labor and delivery) nurses, and the smallest proportion of nurses receiving PMTCT training were postpartum nurses. These differences by specialty unit were significant (Fisher's exact test, *P* = 0.007).

### 3.2. Bivariate Analysis of Training in PMTCT with Demographic Variables

Earlier studies examining HIV knowledge and practice in nurses have demonstrated significant relationships between practical knowledge and nursing experience, professional rank, nursing education, level of care (primary, secondary, and tertiary), and specialty unit type. PMTCT training was significantly associated with age, nursing experience, and specialty unit; however, no significant associations were noted with professional rank, nurse educational level, and facility level of care (primary, secondary, and tertiary). See Table 3.

### 3.3. PMTCT Care Practice Scores

In this group of nurses, the PMTCT care practice scores had a mean of 8.3 (SD 2.6) and ranged from 0–12, with higher scores associated with increased application of PMTCT practices. As illustrated in Table 4, on average, the PMTCT care practice scale scores were higher for nurses who received training in PMTCT, with a mean of *M* = 8.7 (SD 2.0) compared to those without training, with a mean of 7.9 (SD 2.5). The bivariate linear mixed model showed that the PMTCT care practice scores of the group of trained nurses were significantly higher than those of the nontrained group. The care practice scores were highest for nurses working in maternity/labor and delivery and special care baby units (SCBU), with means of 8.4 and 8.6, respectively. The scores were lowest for nurses working in primary health care settings, mean = 7.1 (SD 1.5). These differences by specialty unit were not statistically significant when analyzed in the bivariate linear mixed model (with ANOVA, Wald *χ* ^2^ = 10.40, *P* = 0.109).

### 3.4. Facility Level Variations in Nurse's Knowledge of PMTCT

Nigeria's health system is comprised of three levels: primary, secondary, and tertiary. HIV care was initially centralized in tertiary settings; however, the National Strategic Plan for 2010–2015, developed by the Nigerian National Agency for the Control of AIDS [21], identified decentralization of HIV services, including PMTCT, into the primary settings, as the core strategic approach to improving HIV-related outcomes. Due to this phased rollout approach, nurses in primary and secondary settings had not benefited from observability of evidence-based practice related to PMTCT. Therefore, the data were segregated by level of care (primary, secondary, and tertiary), and the sample was further analyzed based on nurse training in PMTCT for group comparisons. The nurses with the highest PMTCT care practice scores were in tertiary centers (accounting for 59% of the scores that were greater than 10). These univariate trends were not significant with bivariate analysis, Fischer's exact tests *P* > 0.05, indicating no significant difference in PMTCT knowledge by the facility's level of care.

On average, the mean PMTCT care practice scores for nurses employed in primary health care settings were lower, with mean = 7.7 (SD 1.9), than for secondary settings, mean = 8.0 (SD 2.8), or tertiary settings, mean = 8.1 (SD 2.5). These differences by level of care were not significant (Wald *χ* ^2^ = 0.22, *P* = 0.896). Due to their increased experience in HIV care, nurses in tertiary centers were expected to have increased knowledge related to maternal intrapartum treatment. However, when nurses were stratified by the facility's level of care, 37% of tertiary nurses incorrectly responded that they would not provide maternal intrapartum treatment, compared to 25% of primary care nurses and 21% of secondary care nurses. See Table 4.

### 3.5. PMTCT Knowledge Gaps: Individual Item Analysis

Most nurses were able to correctly access maternal HIV laboratory reports, wear gloves, and provide newborns with ART prophylaxis, and many indicated that they would treat mothers with ART during the intrapartum period. It is notable that very few nurses (2.3%) reported being completely unsure about how to treat HIV-positive mothers and their exposed newborns. Most nurses, 91% of trained nurses and 77% of nurses not exposed to PMTCT training, reported correctly that they would provide ART prophylaxis to HIV-exposed newborns. On the other hand, several appropriate evidence-based practices were not reported as being implemented in the clinical setting. Most nurses incorrectly reported they would not bath HIV-exposed infants after birth, recommend breastfeeding to HIV-exposed infants, or wear gowns with every patient. Only 11.8% of nurses who received training in PMTCT reported that they would bath the infant after birth compared to 8.4% of nurses who did not receive training. Most nurses in this study (85.5%), regardless of exposure to PMTCT training, incorrectly reported that they would not recommend breastfeeding by HIV-positive mothers. A significant number of nurses, 27%, incorrectly responded they would not provide antiretrovirals to mothers during the intrapartum period. This gap persisted even for the nurses who received PMTCT training and those in tertiary centers. When asked about universal infection prevention practices in newborns and mothers, 23% of maternal child health nurses reported that they did not wear gloves, and 62% did not wear gowns. Gaps in practices related to universal precautions and lack of utilization of gloves and gowns may increase the risk of contraction of HIV and compromise the safety of mother, newborns, and nurses.

### 3.6. Multilevel Modeling to Account for Clustering of Nurses

Finally, multilevel modeling analysis was performed to evaluate the influence of these nurse and facility characteristics on PMTCT care practices. Under the null model, the intraclass correlation (ICC) was 0.17 (95% CI: 0.044, 0.30) [22]. The random intercept model was appropriate to account for clustering of nurses within the participating facilities. The final model of PMTCT care practices scores was also controlled for nurses' age, years of experience, and level of care (primary, secondary, and tertiary). As summarized in Table 5, age, experience, and level of care were not significant predictors of PMTCT knowledge scores. However, training in prevention of maternal to child transmission was a significant predictor of PMTCT care practices (*b* = 0.7, 95% CI: 0.07, 1.3; *P* = 0.03). On average, nurses who received training had scores 0.7 higher than those without training.

## 4. Discussion

Research evidence related to caring for HIV-infected mothers and HIV-exposed newborns is rapidly evolving (WHO, 2010). Significant progress has been made over the last decade in the global scale-up of prevention of maternal to child transmission of HIV in developing countries; yet very little is known about the actual translation of this knowledge into nursing practice. The current study addresses several important concerns: first, HIV-infected women and their infants deserve the best possible nursing and medical care to break the cycle of the HIV epidemic; second, nurses need to be provided with the knowledge, skills, and attitudes that can help them provide the basic provisional services. Even though nurses are foundational to the healthcare system in Nigeria, their capacity is weakened by lack of sufficient access to the education, practical experiences, and coaching support needed to implement evidence-based practices. The current study identified specific gaps in key PMTCT practices related to maternal intrapartum treatment and breastfeeding in the context of HIV. By contrast, areas where nurses have demonstrated strong knowledge and application of PMTCT practices have also been identified, specifically, their knowledge related to providing antiretroviral treatment (ART) for newborns after delivery. A particular strength of the current study is that all participating facilities maintain a strong ongoing relationship with the Institute of Human Virology-Nigeria (IHV-N), which continues to provide support and training in HIV/AIDS and PMTCT treatment and care.

This study also has some limitations. It utilizes a cross-sectional design, which reflects data collected at only one point in time. In addition, the data in this study were collected while some facilities were in the midst of decentralizing HIV care from tertiary to primary and secondary sites, which may have subsequently altered the level of training available at sites where HIV/AIDS care was previously limited. Furthermore, PMTCT translation was examined in MNCH nurses who were employed in public, government-owned hospitals, and the findings should not be generalized to nurses in the entire Nigerian healthcare system, which has a significant private sector. More generally, the challenge associated with examining the translation of PMTCT knowledge into practice in resource-limited countries is exacerbated by the limited research performed in this area to date.

### 4.1. Translation and Theory

The diffusions of innovation theory [18, 19] and its five determinants of adoption of new evidence-based practices (i.e., relative advantage, compatibility, complexity, trialability, and observability) are applicable to the study results. (See Table 2.) Access to antiretrovirals at all sites, due to the organizational relationships with a donor organization, is a relative advantage and it was expected that it would affect the provision of antiretrovirals in the clinical setting. For newborn prophylaxis, this proposition was supported; however, despite access to maternal antiretrovirals, nurses reported not providing intrapartum ARVs to mothers. Due to the centralization of HIV care in tertiary centers in Nigeria, the lack of observability (not having the opportunity to see the performance in their institutions) may have outweighed the benefit of relative advantage. Nurses in primary and secondary care had limited or no opportunities to observe the practice of caring for HIV mothers. Under the centralized model, once pregnant women were identified as HIV positive, they were referred to a tertiary setting. Currently HIV and PMTCT care and treatment are being decentralized into primary settings; based on the findings in this study, increased training in PMTCT is needed in order for nurses in these settings to perform the appropriate nursing practices to prevent maternal to child transmission of HIV. Nurses working in primary and secondary settings had lower mean PMTCT practice scores than nurses working in tertiary settings; trained nurses had higher mean scores than did nontrained nurses. Surprisingly, nurses in tertiary centers also lacked application of knowledge related to maternal intrapartum care and additional training and may benefit from continued education and training. The failure of nurses to treat mothers with antiretrovirals during delivery is a significant gap in practice that warrants further investigation.

Gaps in practice were also noted with nursing practice related to breastfeeding in the context of HIV. The relative advantage and complexity associated with HIV-positive mothers providing breast milk to their infants were evident in the study. Participating nurses seemed to be bound to the idea that avoidance of breast milk is best and continue to perform duties based on old evidence, indicating a lack of relative advantage. The new evidence (recommending breastfeeding) is not perceived as better than the idea that came before it (avoiding breastfeeding). Secondly, due to the rapid increase in research and change in information on PMTCT, the new evidence related to breastfeeding is complex and appears to be constantly changing. Evidence-based practices related to breastfeeding in the context of HIV have fluctuated greatly over the last decade, thereby muddling practical knowledge and intensifying complexity in the diffusion of research to practice. During the emergent response to the HIV epidemic, the strategy was to avoid breast milk feeding, where possible, in all HIV-exposed newborns [23]. Yet, the social and economic disparities and variations in cultural practices in resource-limited countries made this approach increasingly challenging. Later studies in resource-limited countries demonstrated that the reductions in HIV transmission from replacement feedings were often offset by increases in mortality due to respiratory and diarrheal illness [24]. By 2001, the WHO recognized the increases in mortality and morbidity unrelated to HIV among HIV-exposed infants and [25] introduced the acceptable, feasible, affordable, sustainable, and safe (AFASS) criteria. Each mother was expected, with appropriate counseling support (often delivered by frontline nurses), to use the AFASS criteria in making the decision of whether to use breast milk or replacement milk. More recently, infant feeding guidelines from WHO initiated in 2010 [1] did not use the AFASS language and shifted the decision making away from nurses, counselors, and mothers onto national health authorities, who are expected to provide leadership and guidance by deciding, promoting, and supporting appropriate feeding practices for their country and context.

Frontline nurses have been noted in earlier research to provide “strong advice” rather than counseling on infant feeding decisions in the context of HIV, and therefore may have a direct impact on infant feeding choice at time of discharge [14]. Most nurses in this study would not recommend breast milk and preferred to offer replacement feedings, despite evidence that cost and culture may limit the family's ability to provide replacement formula. This may be grounded in an ethical dilemma related to the evidence-based knowledge that continued infant exposure through breastfeeding still carries an HIV transmission risk between 5–20% [26]. This gap in advocacy for breastfeeding may account for the nearly 72% of women that are HIV positive who decide to use formula/replacement feeding at time of discharge from a Nigerian health facility [3, 27]. However, longitudinal studies have demonstrated that most newborn infants (71%) that were formula fed at time of hospital discharge subsequently switched and were exclusively breastfed at 1 month and 6 months [27]. Switching from replacement formula to breast milk results in exposure to mixed feeding, defined as the combination of replacement milk and breast milk. In recent Nigerian studies, HIV-exposed infants that received mixed feeding were nearly six times more likely than infants that received exclusive formula feeding to acquire HIV [27]. Multiple studies have demonstrated that mixed feeding exposure is associated with a higher risk of transmission of HIV compared to exclusive breastfeeding or exclusive replacement formula [3, 27, 28]. This evidence needs to be translated into clinical practice, and nurses need to take practical steps to prevent new pediatric infections due to maternal to child transmission through breast milk.

Facilitating changes in practice can begin with educating frontline workers about the evidence-based knowledge. This information can be utilized to support such actions as (1) advocating for exclusive breastfeeding, if possible; (2) supporting families' decisions to exclusively use formula feed; and (3) alerting families to the increased risk of acquiring HIV associated with mixed feeding. If nurses are presented with an adequate and continuous flow of research evidence and information, long-standing practices can be changed and gaps in practice can be resolved. Given that training in PMTCT increases knowledge and application of PMTCT practice, nurse training and education are needed to investigate and address this serious gap between knowledge and practice related to breastfeeding of infants exposed to HIV. Nurse education at the preservice level (embedded into their basic nursing education) can advance knowledge of new nurses entering practice, while in-service training (practice-related educational updates) is urgently needed for nurses who are already in clinical practice. Upscaling of nurse education in general to the bachelor's, master's, and doctoral level may improve the dissemination and implementation of research into practice. Although there is currently momentum to advance nursing education to the bachelor's level in Nigeria, in 2009 75% of practicing RNs in Nigeria were certificate or diploma prepared [20].

Discussing the gaps in translation of evidence-based practices in PMTCT using the diffusions of innovation theory provided a basic framework for clearly articulating the challenges around adoption of the practices. Future studies examining the translation of evidence-based practices should also expand on this process by collecting specific, objective data related to nurse perceptions of relative advantage, compatibility, complexity, trialability, and observability. Additionally, using the theoretical framework further assisted in the development of strategic recommendations for improved translation of PMTCT research to practice.

### 4.2. Strategic Recommendations for Improved Translation

The findings in this study could be used to strengthen nurses' ability to provide evidence-based care for HIV-infected mothers and newborns. With the growing emphasis on the translation of research from policy to practice, strategic approaches grounded in implementation science theories and frameworks are needed to increase translation and the rate of adoption for evidence-based practices in PMTCT. Researchers, policymakers, and clinicians should work together to support expedited translation of evidence into real clinical practice. Nurses, in particular, have an important role in providing both practical and scientific insight into the development and implementation of strategies that support translation in both global and national practice settings.

In order to translate research successfully into practice, nurses must first understand why these gaps in translation of evidence into practice remain even in the midst of this surge of research and policy-based guidelines in PMTCT. Secondly, nurses must determine what practical measures can be utilized to address this gap in an era in which research, policy, and practice in PMTCT are rapidly changing. It has been argued that failures to translate evidence into practice remain, in part due to tradition-driven health practitioners, including nurses, who prefer to continue to practice with outdated knowledge as well as researchers and policymakers that believe simply publishing the evidence will result in practical use [29]. Importantly, to a certain extent, PMTCT research has been translated into policy and expert guidelines, as evidenced in both national policies and global policies from the WHO for PMTCT. However, if nurses are to fully realize the application of these evidence-based practices in the clinical setting, additional strategic efforts are needed to ensure that healthcare professionals on the frontline are able to apply the knowledge in practice and have continuing access to new knowledge.

One practical strategic approach is the development of implementation teams that focus on quality, integration, alignment, problem solving, and sustainability of new research knowledge as it enters practice (National Implementation Research Network, 2011). Development of hospital-based implementation teams that are closely aligned with the Ministry of Health (MOH) may increase organizational capacity to update nurses and other healthcare workers regularly on changes in research, policy, and practice. These teams would also provide the structure to support organizational capacity to sustain PMTCT interventions strategically. The interdisciplinary team could be responsible for training nurses on PMTCT practices, continuing to engage them through coaching as well as by modeling the evidence-based interventions in clinical practice, and assisting in the development of feedback mechanisms that address barriers to care. The team could also engage in the complementary process of “exnovation,” which targets false beliefs and outdated practices [29]. This would ensure that in addition to the translation of the new best practices, outdated nursing practices, such as those associated with PMTCT care in Nigeria, would be removed from nursing practice [29]. The team would support connectivity between the Nigerian Federal Ministry of Health (and other key stakeholders) and healthcare facilities to ensure that the best and current evidence-based practices related to PMTCT reach frontline workers. This strategy has the potential to ensure that the national message developed by the Nigerian FMOH on infant feeding practices in the context of HIV reaches and is implemented in “real” practice settings. Furthermore, regular monitoring and evaluation of PMTCT programs for accuracy and consistency by ministries of health and other key stakeholders from nongovernmental organizations, the Nursing and Midwifery Council, nursing schools, and other regulatory bodies could also ensure a system of accountability related to the translation of knowledge into practice. This strategy would ensure that researchers, policymakers, and frontline practitioners work in a complementary way to transfer new knowledge into practice efficiently. These combined efforts serve to advance translation of knowledge to action and improve patient outcomes.

## 5. **Conclusion**

This study provides insight into the translation of evidence-based knowledge related to PMTCT into practice. Nurses demonstrated that some evidence-based interventions related to PMTCT are translating from research and policy into clinical settings; yet significant gaps in key practices remain. The PMTCT knowledge and application gap was found to be ameliorated with training. Innovative strategic interventions to link research, policy, and practice that are grounded in implementation science theory may support efficient translation of PMTCT and nursing research. Ministries of health, program administrators, clinical leaders, and policymakers could apply the evidence and recommendations to improve the content of training programs and to develop novel approaches to ensure translation of research into practice.

## Figures and Tables

**Table 1:** The role of nursing in preventing maternal to child transmission of HIV.

Preconception	Providing education on safe sex practices, HIV testing, counseling, and treatment
Prenatal	HIV testing and counseling; maternal treatment with antiretroviral (ARV) medication if HIV positive; coordinating care and support for adherence, disclosure, and other psychosocial needs; and assistance in navigating a fragmented healthcare system for PMTCT services
Perinatal	Safe delivery practices; intrapartum antiretroviral treatment (ART); immediate neonatal care
Postnatal	Counseling related to informed infant feeding options; continued ART to HIV-positive mothers; and prophylaxis to exposed newborns
Gynecological/family planning	Continued support, treatment, and counseling during the postnatal period related to safe sexual practices and family planning

**Table 2:** PMTCT practice items related to determinants of adoption.

Determinants of adoption	Definition	Related PMTCT practice category	Hypothesized outcomes
Relative advantage	The new practice is perceived as better than the one that preceded it	Newborn antiretroviral (ARV) treatment	Adoption of the practice related to newborn ARV treatment is expected to be positive due to presence of ARVs at all sites due to relationship with donor agency
		Breastfeeding	Earlier recommendation was the avoidance of breastfeeding. The expectation of the study is that the recommendation to breastfeed is not perceived to be better than avoiding breastfeeding. Adoption is not expected
Compatibility	The practice works well with existing practices and structures	Maternal and newborn ARV treatment	Adoption is expected due to availability of ARVs at all sites
		Universal precautions	Adoption of the practice is not expected due to research evidence of limited material resources, including gowns and gloves
Complexity	The practice is simple and well defined	Breastfeeding	Multiple changes in recommendations over the last decade, varying from avoiding breastfeeding to providing breastfeeding. Adoption is not expected
Trialability and observability	The practice is offered in the clinical setting and nurses have opportunities to observe the practice being delivered in their institution	Maternal intrapartum treatment	Due to centralization of HIV treatment in tertiary settings, nurses in primary and secondary settings had no opportunities to trial or observe care of HIV-positive pregnant women. Adoption of the practice is not expected to be implemented in primary and secondary sites

**Table 3:** Nurse demographic characteristics by PMTCT training, *N* = 223^a^.

MNCH nurse characteristics	PMTCT training *N* = 93 (43%)	No PMTCT training *N* = 123 (57%)	*P* value^†^
	*M* (SD)	*M* (SD)	
Age (years)	43 (8.1)	39.1 (8.8)	0.001
RN experience (years)	18.3 (8.6)	15.2 (8.8)	0.01
Professional rank	*N* (%)	*N* (%)	0.764
Staff nurse, direct care	68 (43)	89 (57)	
Staff nurse, direct & indirect^b^	14 (48)	15 (52)	
Nurse matron	10 (38)	16 (61)	
Nursing education			1.0
Diploma prepared	90 (43)	118 (57)	
BSN or MSc	3 (38)	5 (62)	
MNCH specialty (assigned unit)			0.007^‡^
Maternity (labor and delivery)	37 (52)	34 (48)	
Special care baby unit//neonatal ICU	10 (25)	29 (74)	
Antenatal clinic	19 (61)	12 (39)	
Primary healthcare	4 (44)	5 (56)	
Pediatric unit	10 (33)	20 (66)	
Rotating MNCH nurses	9 (45)	11 (55)	
Postpartum	2 (14)	12 (86)	
Facility/level of care			0.067
Primary	5 (31)	11 (69)	
Secondary	44 (52)	40 (48)	
Tertiary	43 (37)	72 (62)	

^a^ *N* varies due to missing data; ^b^nurses had additional administrative duties; ^†^ *t*-test or chi-square test was used; ^‡^Fisher's exact test was used.

**Table 4:** PMTCT care practice scores by training, level of care, and specialty unit.

	Mean (SD)	Wald *χ* ^2^ (df)	*P* value
Training		7.21 (1)	0.007
Not trained in PMTCT	7.9 (2.5)		
Trained in PMTCT	8.7 (2.0)		
Level of care		0.22 (2)	0.896
Primary	7.7 (1.9)		
Secondary	8.0 (2.8)		
Tertiary	8.1 (2.5)		
Specialty unit		10.4 (6)	0.109
Antenatal clinic	8.3 (2.8)		
Maternity (L&D)	8.4 (2.7)		
Special care baby unit (SCBU)/neonatal ICU	8.6 (2.2)		
Pediatric unit	7.3 (2.7)		
Rotating MNCH nurses	7.6 (2.2)		
PHC setting	7.1 (1.5)		
Postpartum	7.2 (2.9)		

The *b* (95% CI) and *P* value were estimated from the bivariate linear mixed models of PMTCT knowledge score on each predictor separately. A random intercept of facility was included in each model to account for clustering of nurses within facilities.

**Table 5:** Association between PMTCT training and PMTCT care practice using a linear mixed model.

	*b* (95% CI)^†^	*P* value
Age (years)	−0.003 (−0.08, 0.08)	0.095
RN experience (years)	0.01 (−0.07, 0.09)	0.805
PMTCT training (yes versus no)	0.7 (0.07, 1.3)	0.03
Type of facility		
Secondary versus primary	0.6 (−0.8, 2.0)	0.4
Tertiary versus primary	0.4 (−1.0, 1.7)	0.619

^†^Linear mixed model adjusted for age, experiences, and type of facility and with a random intercept of hospital to account for clustering of nurses within each hospital.

## Abbreviations

AFASS: Acceptable, feasible, affordable, sustainable, and safe feeding criteria
ART: Antiretroviral treatment
ARV: Antiretroviral
ARVs: Antiretrovirals
EBP: Evidence-based practices
FMOH: Nigerian Federal Ministry of Health
ICU: Intensive care unit
IHV-N: Institute of Human Virology Nigeria
MCH: Maternal child health
MOH: Ministry of Health
MNCH: Maternal, newborn, child health
MTCT: Maternal to child transmission
PMTCT: Prevention of mother to child transmission
RN: Registered nurses
SCBU: Special care baby unit
TBA: Traditional birth attendant.
